# Rhizoremediation of Cu(II) ions from contaminated soil using plant growth promoting bacteria: an outlook on pyrolysis conditions on plant residues for methylene orange dye biosorption

**DOI:** 10.1080/21655979.2020.1728034

**Published:** 2020-02-17

**Authors:** P. R. Yaashikaa, P. Senthil Kumar, Sunita Varjani, A. Saravanan

**Affiliations:** a Department of Chemical Engineering, SSN College of Engineering, Chennai 603 110, India; b Gujarat Pollution Control Board, Gandhinagar, Gujarat, India; c Department of Biotechnology, Rajalakshmi Engineering College, Chennai, India

**Keywords:** Metal resistant bacteria, *pantoea dispersa*, phytoremediation, pyrolysis, biochar

## Abstract

Rhizoremediation is one of the most accepted, cost-effective bioremediation techniques focusing on the application of rhizospheric microorganisms in combination with plants for the remediation of organic and inorganic pollutants from the contaminated sites. This work focuses on isolation and identification of metal resistant bacteria to grow on medium with the copper ion concentration of 1500 mg/L. The resistant isolate was identified as *Pantoea dispersa* by a 16S rRNA sequencing. The bioaccumulation of Cu(II) ions in plant is high at the concentration of Cu(II) ion is 125 mg/L in soil. In *Sphaeranthus indicus* the Cu(II) ion translocation factor has expanded with an expansion of grouping of Cu(II) ion in the soil and the most extreme TF factor was acquired at the centralization of Cu(II) ion is 150 mg/L in soil. Surface morphology of biochar was characterized by Scanning Electron Microscopy (SEM) analysis. The adsorption performance of biochar (*Sphaeranthus indicus* biomass) and mechanism for the removal of Cu(II) ion were investigated. This study resolves that pyrolysis is promising technology for the conversion of metal ion contaminated plant residues from phytoremediation into valuable products.

## Graphical Abstract

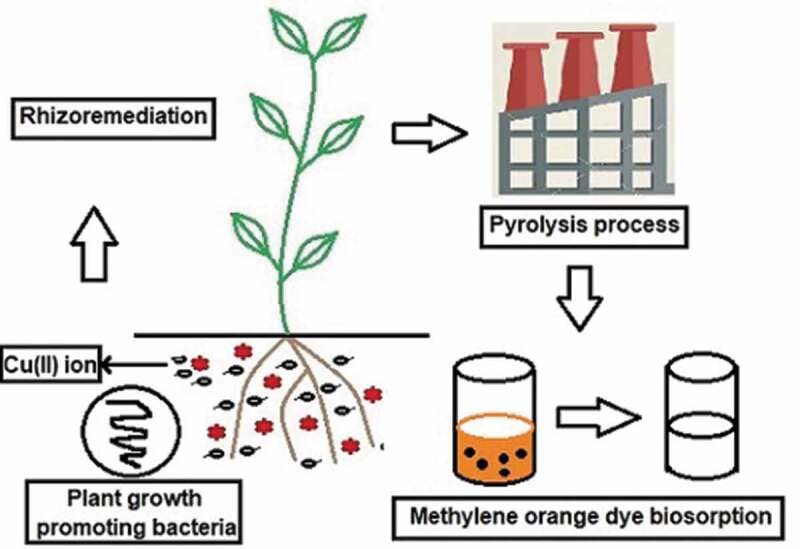

## Introduction

The contamination of toxic heavy metals in the soil makes a severe risk to humans and environment on account of its non-biodegradability and poisonous quality [1,2]. The soil contamination by poisonous heavy metals has quickened significantly by the utilization of heavy metals, for example, chromium, cadmium, zinc, lead, mercury, copper and nickel [3–5]. These toxic heavy metals are directly introduced into the environment through the rapid development of different industries such as metal plating, fertilizer, battery, mining, paper, pesticides, electroplating, tannery, leather, iron and steel [6–8]. In this manner, remediation of toxic heavy metals is important to shield the earth from their lethal impacts. A few endeavors have been made to create manageable and environmental friendly advancements valuable to remove and expel dangerous heavy metals from water and soil [9–11].

These heavy metal contamination causes several health effects to humans such as may damage liver, lungs, kidney, nervous system, headache, anxiety and depression, digestive problems, autoimmune diseases, vomiting, muscle weakness [12–16]. Different treatment technologies have been used for the removal of toxic heavy metals such as ion exchange [17], membrane separation [10], reverse osmosis [18], electro chemical treatment [19], precipitation [20], chelation [21], electro dialysis [22] and ultrafiltration [23]. These ordinary advances utilized for the removal of toxic heavy metals from profluent wastewater have a few disadvantages, for example, high establishment cost, tedious running, costly upkeep, low productivity of execution at low metal concentration and issues with transfer of sludge. There is an important to stop the gathering of these natural poisons and expels them from the earth without influencing different assets [24,25]. In the substitute, organic approaches/bioremediation is considered as the best, eco-accommodating and minimal effort strategy for metal remediation without changing the physiochemical properties of the dirt.

The remediation procedures to beat the issues of environmental degradation ought to be permeated for its environmental effects. As of late, phytoremediation is viewed as a noticeable innovation for the expulsion of toxic contaminants. Phytoremediation is a pertinent strategy to a few recovering treatment, since it does not meddle with the biological system, it requires little manpower and accordingly is not extravagant contrasted with customary physicochemical techniques. Phytoremediation strategies could be applied for the recuperation of the modern destinations vigorously debased. Plant rhizosphere associations with contaminants, are the key factor in phytoremediation innovations. Plants take up the metals from the rhizosphere as pursues: Mobilization of heavy metals in soil and ensuing take-up by plant roots, translocation of the collected metals from roots to airborne tissues pursued by sequestration of the metals in plant tissues and resistance [26–31].

The achievement of phytoremediation is reliant on the capability of the plants to yield high biomass and withstand the metal pressure. Moreover, the metal bioavailability in rhizosphere soil is viewed as another basic factor that decides the productivity of metal translocation and phytostabilization process. Despite the fact that these changes increment the productivity of phytoextracton/phytostabilization, some concoction alterations are not just phytotoxic yet additionally dangerous to gainful soil microorganisms that assume significant job in plant development and advancement [32–34]. Bioremediation is characterized as the procedure whereby toxins naturally lessen under controlled conditions to a harmless state, which comprise of phytoremediation, biopile, windrows, bioreactor, land cultivating, bioventing, bioslurping, biosparging, penetrable responsive boundary, bioaugmentation, bioleaching and treating the soil. Among them, phytoremediation is the more dominant and broadly acknowledged system for metal remediation. A promising option in contrast to concoction changes could be the utilization of organism intervened forms, in which the microbial metabolites/forms in the rhizosphere influence plant metal take-up by changing the versatility and bioavailability [35,36].

When thinking about ways to deal with adjusts heavy metal mobilization, there are a few preferences to the utilization of favorable organisms as opposed to substance changes on the grounds that the microbial metabolites are biodegradable, less poisonous and it might be conceivable to create them in situ at rhizosphere soils [37–39]. Building pyrolysis innovation for feasible administration of contaminated biomass coming about because of phytoremediation and their use as the feedstock for synchronous generation of significant items requires examination of the biomass warm conduct. This examination meant to explore the impact of pyrolysis temperature (500–800°C) on the properties of polluted biomass resulted from phytoremediation and resulting pyrolytic products. *Sphaeranthus indicus* acquired after phytoremediation of Cu(II) ions smelter slags was utilized as a model biomass. The outcomes were additionally contrasted with an uncontaminated *Sphaeranthus indicus* biomass to decide the impact of heavy metals on the properties of the resultant pyrolytic products.

In this study, *Sphaeranthus indicus* plant has been chosen and the Cu(II) ion resistant bacteria—*Pantoea dispersa* was isolated from the tannery-effluent exposed area. This crop is being created in the zone adjoining the organizations where current effluents spoiled with significant metals is heading off to the field so the present assessment is grasped to think about the phytotoxic effects of Cu(II) ion on advancement parameters in *Sphaeranthus indicus*. With this foundation, the present investigation planned to recognize the phytoremediation capacity of *Sphaeranthus indicus* in Cu(II) ion tainted soil with the plant growth developing microorganism—*Pantoea dispersa*. The aim of the present study was (i) to identify the potential of *Sphaeranthus indicus* plant with the integrated approach for the enhanced phytoremediation of Cu(II) ions from the soil (ii) to isolate the Cu(II) ion-resistant bacteria and identifying the isolated bacteria by 16s rDNA sequencing and phylogenetic tree analysis (iii) to analyze the metal degrading ability of *Pantoea dispersa* by biodegradability assay (vi) to investigate the growth development of *Sphaeranthus indicus* plant coupled with *Pantoea dispersa* in the Cu(II) ion-contaminated soil (v) to estimate the translocation and bioaccumulation factor of *Sphaeranthus indicus* plant (vi) to characterize the *Sphaeranthus indicus* biomass proliferate with Cu(II) ions after phytoremediation (vii) to estimate the biosorptive applications of biochar (*Sphaeranthus indicus* biomass) for the removal of methylene orange dye from aqueous solution.

## Materials and methods

### Copper resistant bacteria

The soil samples were collected from the Vaniyambadi region in Vellore district, Tamil Nadu, India. This region has been exposed to the large number of toxic tannery industrial wastes. The soil samples were transferred to the laboratory by using sterile glass bottles and allowed for serial dilution (10^−1^ – 10^−9^) by using the distilled water. The dilutions of 10^−4^, 10^−5^ and 10^−6^ were plated (streak) by using Nutrient Agar (NA) for the isolation of heavy metal resistant bacteria. After plating they were allowed to incubate at 30°C for 48 h. After the incubation different morphological colonies were absorbed in the plate, from that specific colony were isolated and inoculated into another NA plate for the isolation of specific species from the group of colonies, this process will occurs until the homogenous growth phase is obtained. The isolated bacterial samples were allowed for sequencing study and phylogenetic analysis, which was performed in Yaazh Xenomics, Chennai.

### Sequencing

For the sequencing study of isolated bacterial species, the DNA extraction was done by using the standard procedure. The concentration of DNA was measured by running aliquots on 1% agarose gel. Using PCR (Polymerase Chain Reaction) specific resistant gene was amplified after isolation process. PCR was executed using two different primers (Forward and Reverse primer) both are having 20 bp named as 25F and 1492R and their sequences are; 5ʹ AGAGTTTGATCMTGGCTCAG 3ʹ and 5ʹ TACGGYTACCTTGTTACGACTT 3ʹ respectively. 5µL of isolated DNA was mixed with 25 µl of PCR reaction mixture containing the primers, Taq Master Mix and deionized water. Using five different thermal conditions the reaction was achieved, the thermal conditions are; Initial Denaturation (94 °C, 3 min), Denaturation (94°C, 30 s), annealing (60°C, 30 s), extension (72°C, 1 min) and Final extension (72 °C, 10 min). Finally the mixture were hold at 4°C, using single-pass sequencing the developed PCR results were molecularly characterized (sequenced) and an amplified outcomes were subjected to electrophoresis at Yaazh Xenomics, Coimbatore, Tamil Nadu, India.

### Phylogenetic analysis

The phylogenetic examination with other firmly related grouping was done trailed by various succession arrangements utilizing the program MUSCLE 3.7. The precise Phylogenetic investigation was performed utilizing the program PhyML 3.0.

### Biodegradability assay

The isolated bacteria, *Pantoea dispersa*, was sub cultured in LB broth medium and kept in shaking incubator for the period of 24 h at the temperature of 37 °C and pH of 7.0. Biodegradability assay was performed by adding 100 mL of different concentration of Cu(II) ion into the 250 mL of Erlenmeyer flask containing LB broth medium. The flasks were kept in temperature controlled shaking incubator at the temperature of 37°C for about 24 h. After the mentioned time period, the culture was taken and centrifuged at 5000 rpm for the period of 15 min. Supernatant was isolated and blended in with equivalent amount of concentrated nitric acid followed by heating at the temperature of 100°C until introductory volume of supernatant was acquired. At that point the supernatant is filtered utilizing Whatman filter paper and the concentration of Cu(II) ion in the solution was examined utilizing Atomic Adsorption Spectroscopy (AAS).

### Plant growth analysis and percentage removal of Cu(II) ions

The sieved soil was filled in the six distinct pots each as 500 g and washed with refined water. The various focuses (25–150 mg/L) of arranged CuSO_4_ solution were included into the five pots separately aside from the control. The bacterial detach was immunized in the Luria Broth (LB) and kept in incubator at 30°C for 24 h. The cell suspension were changed in accordance with Optical Density at 600 nm (OD_600_) between 1.4 and 2.0, after that the three sound, comparative size and shape seeds of *Sphaeranthus indicus* seeds per pot were planted and permitted to develop. Subsequent to planting the seeds 20 mL of brooded and upgraded bacterial culture *Pantoea dispersa* were vaccinated into the each pot. Following 45, 55 and 65 days of development the plants were collected and their wet and dry loads are noticed, the roots, shoots and leaves of the plant were ground.

The length and weight of the shoot (stem and leaf) and roots were evaluated. The plant materials were kept in oven at 70°C for 24 h and thereafter dried materials were ground using mortar and pestle. At that point the dried material was permitted to corrosive absorption by the accompanying method. The grounded plant matter was handled using 5 mL of destructive mixes (60% HCl and 85% HNO_3_) at medium-term. After the endorsed time interim, the examples were viewed as cooling and 2.5 mL of 20% HCl was added to the cooled tests, mixed inside and out and warmed at 80 °C for 30 min. The warmed examples were cooled and separated through Whatman 42 channel paper. The concentration of Cu(II) ion in the dirt was assessed when the phytoremediation and moreover in the plant materials (shoot and roots) using Atomic Absorption Spectroscopy (AAS).

### Translocation factor and bioaccumulation factor

Translocation factor (TF) is the extent of centralization of metal in shoot to convergence of metal in root. It is an ability of a plant to translocate the metal from roots through shoots and leaves of a plant which is mindful of Phytoextraction, in like manner called as shoot-root remainder. Cu(II) ion trans-location from root to shoot was evaluated by

(1) $$TF=\;\frac{C_{Shoot}}{C_{Root}}$$

Where, C_shoot_ is concentration of metal in shoot of the plant, C_root_ is concentration of metals in root of the plant.

Bioaccumulation factor (BAF) is the extent of collection of metal in shoot to intermingling of metal in soil. Bioaccumulation is the technique where the poisonous metals are accumulated in different pieces of the plants like shoot and leaves as non-dangerous. Bioaccumulation of Cu(II) particle in plant was determined by

(2) $$BAF=\frac{C_{Shoot}}{C_{Soil}}$$

Where, C_shoot_ is concentration of metal in plant shoot, C_soil_ is concentration of metal in soil.

### Methyl orange (MO) dye solution

In the present study, MO dye solution was used as adsorbate, which was procured from E. Merck, India. A stock solution of 1000 mg/L of MO dye solution was prepared by dissolving required quantity of methylene orange in 1000 mL of distilled water. Then the stock solution was diluted to required concentration (25 to 150 mg/L) using deionized water. The initial and final concentration of MO dye in the solution was analyzed using UV visible spectrophotometer. The pH of the solution was adjusted to neutral using 0.1 N NaOH or 0.1 N HCl.

### Preparation of biosorbent—biochar

The Cu(II) ion contaminated *Sphaeranthus indicus* plant residues (SIPR) has been used as feedstock for the pyrolysis study. *Sphaeranthus indicus* stem were collected and cut into small pieces and washed using pure water to remove the dust particles. The collected stem particles were dried in hot air oven at the temperature of 80°C for about 1 h. The dried stem particles were grinded into fine powder. The grinded fine particles were pyrolyzed at 500, 600, 700, 800°C using a vacuum tube sintering furnace (Manish Scientific Instruments Company, Chennai, India) to produce biochar. The muffle furnace was kept on warming up at the warming pace of 10°C/min until the prescribed temperature was reached. Afterward, the obtained temperature was kept up for 2 h. All the pyrolysis forms were completed with presence of N_2_. After pyrolysis, the materials were cooled to air temperature and put away in a desiccator before use.

### Characterization study

The newly synthesized biochar *Sphaeranthus indicus* plant residue at the pyrolysis temperature of 700°C (SIPR-700) has been utilized as adsorbent material for the removal of methylene orange (MO) dye from aqueous solution. The surface morphology of the adsorbent material plays an imperative role for the determination of adsorption characteristics. The surface morphology of newly synthesized pyrolytic biomass residues was characterized by Scanning Electron Microscopy (SEM) analysis.

### Batch adsorption study

Adsorption of methylene orange (MO) dye was carried out by using the biosorbent material of SIPR-700. Batch experimental study for the removal of MO dye onto SIPR-700 was examined at different factor influencing parameters such as MO dye concentration (25 mg/L to 150 mg/L), contact time (10 to 90 min), pH (3.0 to 10.0), SIPR-700 dosage (0.5 to 2.5 g) and temperature (30 to 60 °C). MO dye adsorption experiments was performed at 100 mL of desired concentration of MO dye solution, pH of 3.0, temperature at 30 °C for about different time interval. After the prescribed time interval, flasks were withdrawn from the shaker and the MO dye solution was filtered using Whatman filter paper. The MO dye concentration in the supernatant was measured by using UV visible spectrophotometer. Every one of the analyses was done in triplicates and the reports were furnished with average of these qualities. The percentage removal of MO dye was determined utilizing the accompanying condition:

(3) $$Percentage\;removal=\frac{C_{i}-C_{f}}{C_{i}}\;x\;100$$

Where C_i_ is the initial concentration of MO dye and C_f_ is the final concentration of MO dye.

## Results and discussion

### Copper resistant bacteria isolation

The resistant bacteria were observed after 24–36 h of incubation. 27 isolates were obtained by serial dilution in the plates 10^−2^ and 10^−3^ with copper ion concentration of 100 mg/L. On further screening, these 27 isolates were grown on nutrient medium plates with high copper ion concentration of 1500 mg/L. Among the 27 isolates, one isolate was grown and shown high resistance at high copper ion concentration. The isolated strain was selected and sub cultured by streak plate method. The plate was incubated at 37°C for 24 h.

### Sequencing

**Figure 1.**
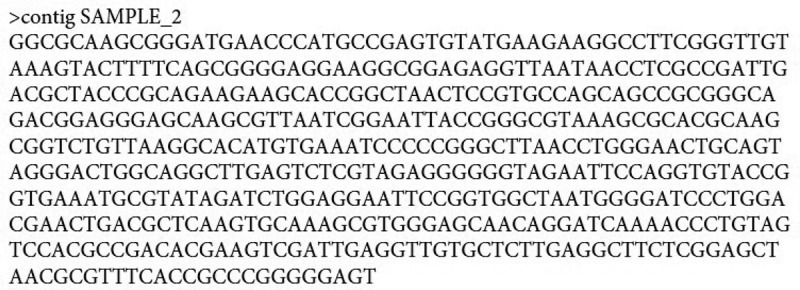
*Pantoea dispersa –* Sequence (Supplementary).

DNA was isolated and the PCR products were purified. Sanger method was used for sequencing the DNA strands. The isolate was found to be *Pantoea dispersa*. Figure 1 represents the 16s rRNA sequence of the isolate. MEGA 6.0 software was used for constructing phylogenetic tree. The 16s rDNA sequence of the bacterial isolate was submitted to NCBI Gene bank. The complete genome sequence of sequenced gene is available in NCBI GenBank under the accession number MH985357.1.

### Phylogenetic tree construction

**Figure 2.**
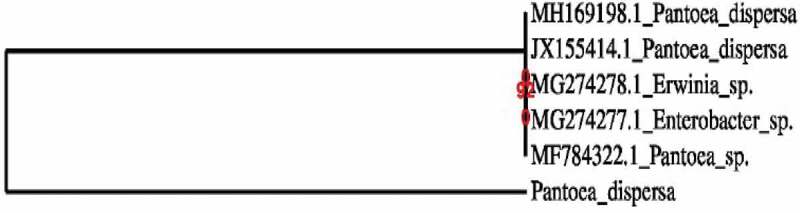
Phylogenetic tree of *Pantoea dispersa.*

Phylogenetic tree was developed utilizing MEGA 6.0 programming for inducing the phylogeny or transformative relationship among other species and to develop branching tree dependent on their similitudes and contrasts in their characters. Figure 2 represents the phylogenetic tree of the resistant isolate. This phylogenetic tree helps in determining the similarity with other species and finding out their similarity and differences in the characters.

### Biodegradability assay

**Figure 3.**
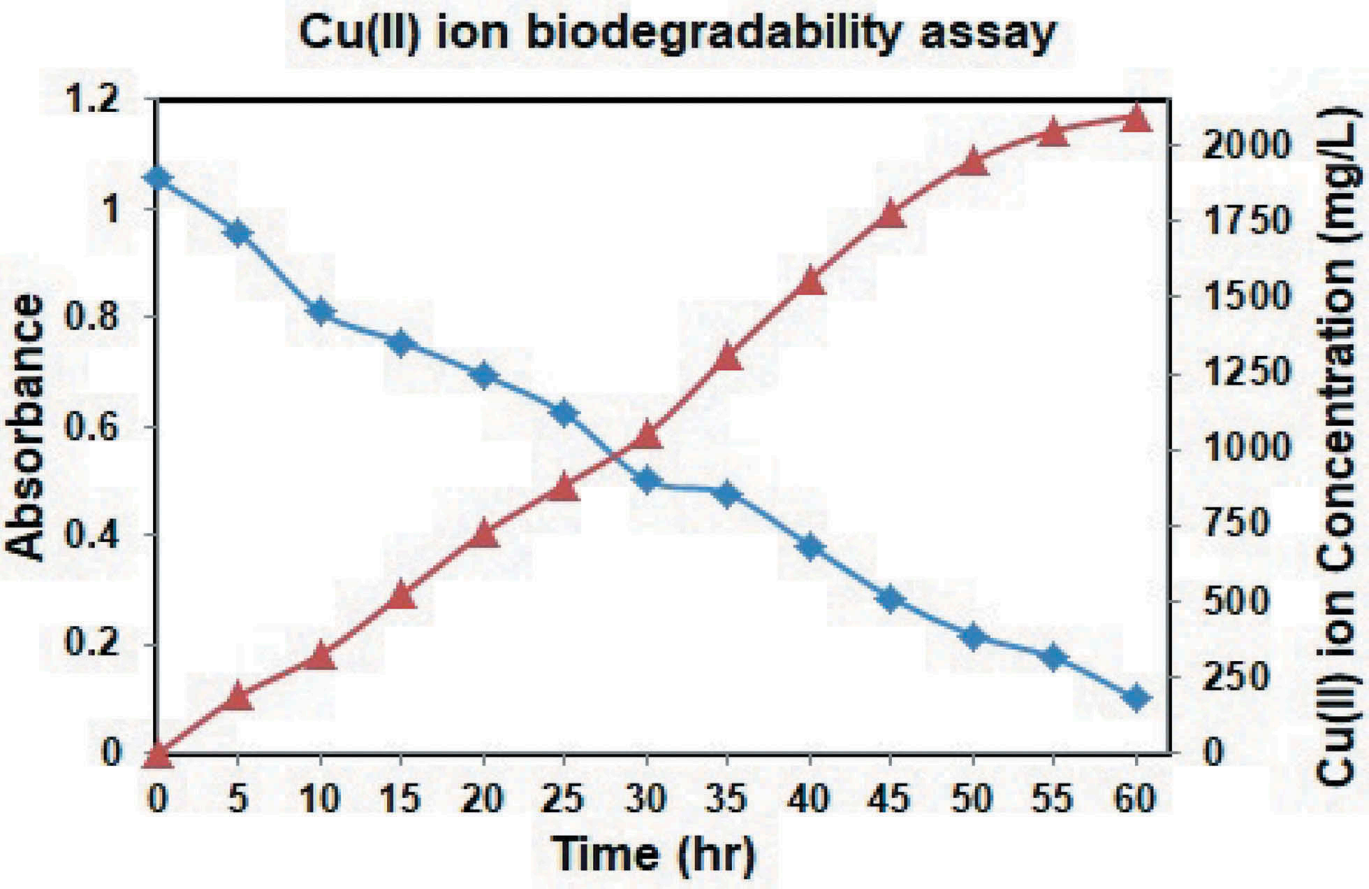
Biodegradability assay.

The sample was incubated and centrifuged followed by the supernatant of isolate was mixed with concentrated HNO_3_. The sample mixture was warmed until essential volume was gotten. At that point the amount of desired Cu(II) ion in the treated supernatant estimated utilizing AAS and the results showed a decline in the Cu(II) ion concentration contrasted with initial Cu(II) ion concentration. The strain can lessen up to the Cu(II) ion concentration of 2100 mg/L. Figure 3 obviously portrays the metal debasing capacity of *Pantoea dispersa*. The bacterium doesn’t display any metal decrease after 2100 mg/L.

### Plant growth analysis and phytoremediation study

**Figure 4.**
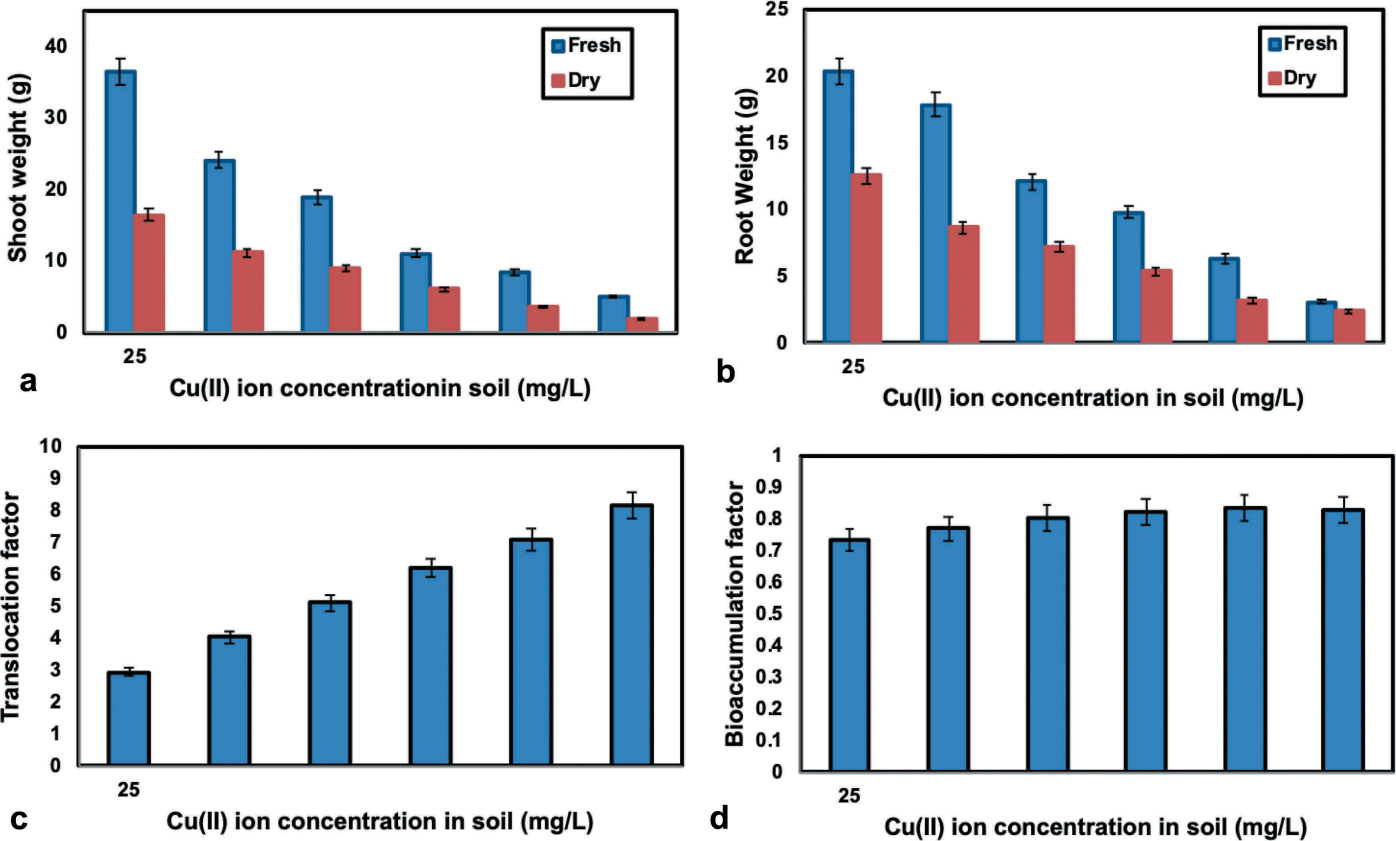
(a) Relation between the shoot weight (*Sphaeranthus indicus*) and Cu(II) ion concentration in soil (b) Relation between the root weight (*Sphaeranthus indicus*) and Cu(II) ion concentration in soil (c) Relation between translocation factor and Cu(II) ion concentration in soil (d) Relation between bioaccumulation factor and Cu(II) ion concentration in soil.

The shoot and root weight were estimated when drying also, the outcomes are appeared in Table 1. The relationship between the Cu(II) ion concentration in soil and shoot weight and root weight was shown in Figure 4(a,b) separately. It was indicated that, shoot weight and root weight (fresh and dry) has bit by bit diminished with an expansion of initial Cu(II) ion concentration from 25 to 150 mg/L. The purpose behind this conduct, Cu was intruding with a factor straight forwardly related with cell prolongation suggesting the specific commitment of synthetic substances in the divider and ATPase related with the plasmalemma.

**Table 1.** Shoot and root – fresh and dry weight of *Sphaeranthus indicus* in soil associated with *Pantoea dispersa* amended with Cu(II) ion concentration.

S. No	Cu(II) ion concentration in soil (mg/L)	Weight of shoot fresh (g)	Weight of shoot dry (g)	Weight of root fresh (g)	Weight of root dry (g)
1	25	36.58	16.47	20.35	12.55
2	50	24.15	11.25	17.84	8.66
3	75	18.95	8.95	12.11	7.18
4	100	11.05	6.11	9.84	5.4
5	125	8.45	3.55	6.33	3.2
6	150	5.01	2.05	3.11	2.4

Phytoremediation of Cu(II) ion from the metal rich soil was done and the results have showed up in the Table 2. From the arrangement report, the results shows that Cu(II) ion in soil were about decreased. The Cu(II) ions were translocated from the dirt to the root and shoot of plant; here they are gathering in the vacuoles. In the vacuoles, they are put away in non-dangerous structure because of detoxification instrument in the vacuoles. From the Table 2, the centralization of Cu(II) ion was found in the root and shoot, in which the grouping of Cu(II) ion is more in shoot of the plant differentiated and root. In perspective on the Cu(II) ion concentration in soil they can be assemble in the shoot and root, when the centralization of Cu(II) ion constructs the collection of Cu(II) ion similarly augments straightforwardly in the shoot and establishment of the plant.

**Table 2.** Cu(II) ion concentration in soil sample and shoot, root (on a dry weight premise) of *Sphaeranthus indicus* associated with *Pantoea dispersa.*

S. No	Cu(II) ion concentration (mg/L)			
	Soil sample (before phytoremediation)	Soil sample (after phytoremediation)	Shoot (*Sphaeranthus indicus*)	Root (*Sphaeranthus indicus*)
1	25	0.289	18.41	6.2
2	50	1.85	38.52	9.53
3	75	3.01	60.25	11.74
4	100	4.57	82.21	13.22
5	125	6.03	104.25	14.72
6	150	10.24	124.56	15.2

### Translocation factor and bioaccumulation factor

The translocation factor (TF) chooses the practicality of Cu(II) ion translocation from root to shoot and the bioaccumulation factor (BAF) arranges the plants as aggregators, hyper accumulators and excluders subject to the congregation of Cu(II) ion. Figure 4(c,d) shows that Translocation and Bioaccumulation factor of Cu(II) ions of *Sphaeranthus indicus* and the results have showed up in Table 3. From Figure 4(c,d), it was seen that Cu(II) ions are successfully translocated from the root to shoot of the plant. The bioaccumulation of Cu(II) ions in plant is high at the concentration of Cu(II) ion is 125 mg/L in soil. In *Sphaeranthus indicus* the Cu(II) ion translocation factor has expanded with an expansion of grouping of Cu(II) ion in the soil and the most extreme TF factor was acquired at the centralization of Cu(II) ion is 150 mg/L in soil. On the off chance that TF > 1, the translocation of metals from the root to shoot is successful. From the organization report, it was indicated that TF and BAF regard more conspicuous than 1, by then it is a great gatherer plant. Fittingly, in the present examination, *Sphaeranthus indicus* goes about as a convincing gatherer on account of its higher TF and its BAF being more unmistakable than 1. Higher gathering of heavy metals in plants shows cut down centralizations of metal, both in the water and the leftovers of the wetland. Thusly, *Sphaeranthus indicus* goes about as a progressively conspicuous phytoremediator of Cu(II) ions in light of its higher translocation limit.

**Table 3.** Translocation and Bioaccumulation factor of Cu(II) ions of *Sphaeranthus indicus* associated with *Pantoea dispersa.*

S. No	Sample	Translocation factor	Bioaccumulation factor
1	1	2.96	0.736
2	2	4.04	0.77
3	3	5.13	0.803
4	4	6.21	0.822
5	5	7.08	0.834
6	6	8.19	0.83

### Characterization study

**Figure 5.**
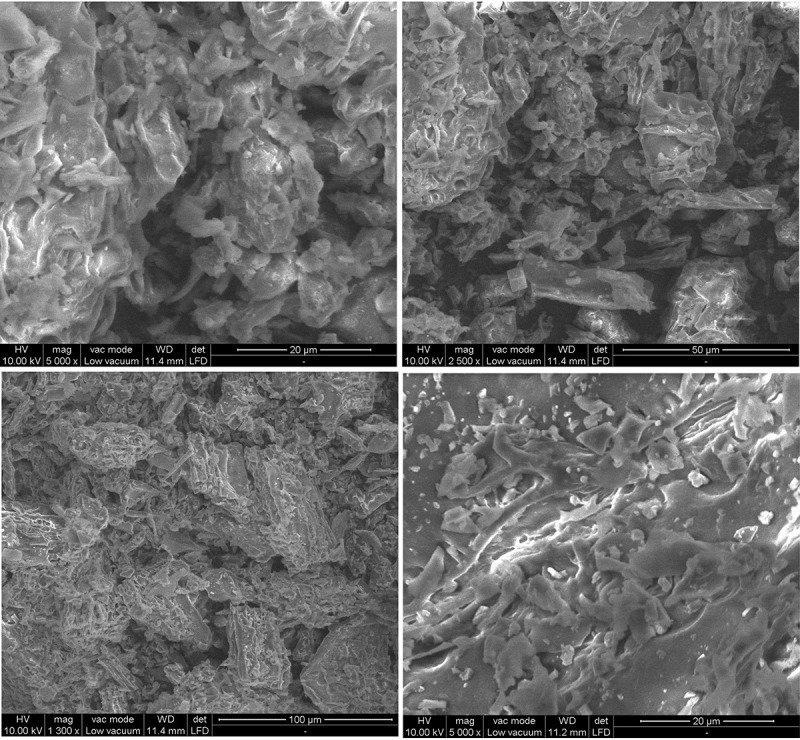
SEM image of *Sphaeranthus indicus* plant residue – Biochar.

The surface structure and morphology of SIPR-700 was characterized by Scanning Electron Microscopy at different magnifications of 1300 x (100 µm), 2500 x (50 µm), 5000 x (20 µm – 11.4 mm) and 5000 x (20 µm – 11.2 mm) and the results were shown in Figure 5. SEM analysis can be used to identify the pores present in the surface of the adsorbent material. Figure 5 shows that SIPR-700 has enormous cavities, more pores and non-permeable solid material as for its surface smoothness. Enormous cavities were formed on the surface of the SIPR-700 due to pyrolysis process. In addition to that, the water molecules present on the pores of the adsorbent material has also been removed by undergoing pyrolysis process. These characteristics indicated that SIPR-700 has more adequate qualities for the removal of MO dye from aqueous solution.

### Batch adsorption study

#### Influence of MO dye concentration

**Figure 6.**
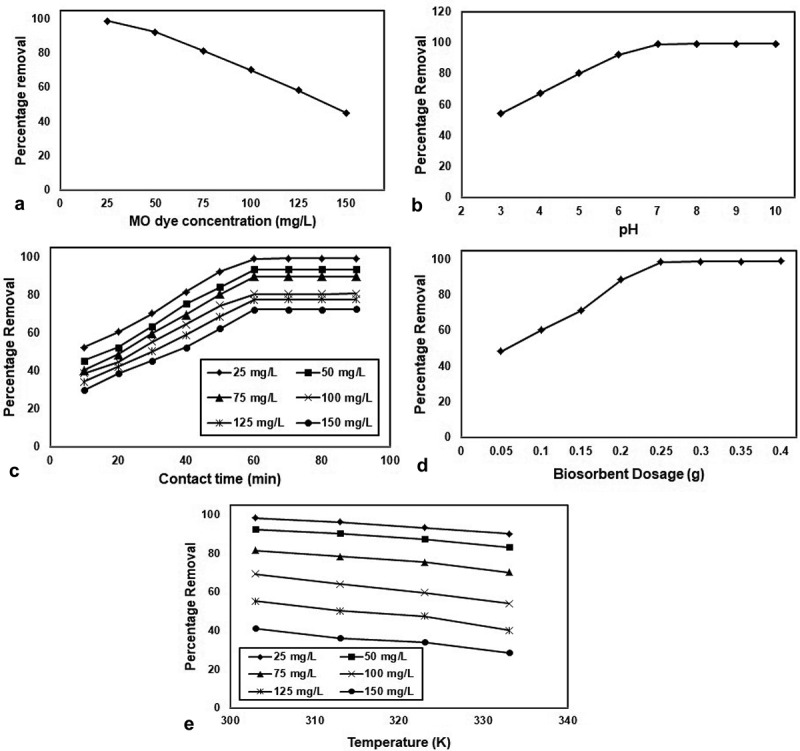
(a) Effect of MO dye concentration for the removal of MO dye onto biochar (b). Effect of pH for the removal of MO dye onto biochar (c). Effect of contact time for the removal of Cu(II) ion onto biochar (d). Effect of biochar dosage for the removal of MO dye onto biochar (e). Effect of temperature for the removal of MO dye onto biochar.

The initial concentration of MO dye assumes a significant job in the adsorption procedure for deciding the adsorption limit of the adsorbent material. In the present investigation, the impact of initial MO dye concentration was examined by varying the MO dye concentration from 25 to 150 mg/L at the pH of 7.0 for 60 min and the biochar dose of 0.25 g at the temperature of 30°C. Figure 6(a) shows the impact of initial MO dye concentration on the adsorption of MO dye onto SIPR-700. It was seen from Figure 6(a), percentage removal of MO dye was diminished with expanding the MO dye concentration from 25 to 150 mg/L. This might be expected to, at lower concentration, MO dye in the fluid arrangement would conceivably bind with the surface dynamic locales of the adsorbent material and along these lines make conceivable practically complete adsorption. In the resistance of, at higher concentration, MO dye are left unadsorbed in the fluid arrangement which may be perhaps because of less accessibility of dynamic locales for example immersion of dynamic locales.

#### Influence of pH

Figure 6(b) shows the impact of pH on the adsorption of MO dye onto SIPR-700 with a wide scope of pH from 3.0 to 9.0. The pH of the MO dye solution was balanced by utilizing 0.1 N HCl and 0.1 N NaOH and estimated by utilizing the pH meter. The adsorption procedure was completed with 100 mL of MO dye concentration (25 mg/L) with various pH (3.0 to 9.0) and biochar measurements (0.25 g) with a contact time of 60 min. As the outcomes from the Figure 6(b), the take-up of MO dye was seen as expanding with increment in the arrangement pH from 3.0 to 7.0. By and large, the MO dye displays the emphatically charged particles when broken down in water. In the pH 3.0 to 7.0, the adsorption of MO dye was expanded; the purpose behind this conduct may be because of essence of an acidic medium, the surface of the adsorbent may get positive charge which doesn’t draw in the decidedly charged MO dye in the solution. As the solution pH increments, the adsorbent surface accomplishes the negative charge which improves the adsorption limit of SIPR-700 due to the arrangement of electrostatic interaction between the adversely charged SIPR-700 and the decidedly charged MO dye. However, further expanding the pH to 9.0, result no change in the adsorption of MO dye on SIPR-700. This conduct can be clarified by at basic medium (pH 7.0) repugnance happens between the adsorbent material (SIPR-700) and the base mechanism of the MO dye solution, for the explanation that, at soluble pH the MO dye in the solution likewise goes to negative charge. Hence, the ideal pH for the evacuation of MO dye onto SIPR-700 was seen as 7.0.

#### Influence of contact time

To explore the contact time for the adsorption of MO dye onto SIPR-700 was performed at various contact times from 10 to 90 min for an underlying MO dye concentration of 25 mg/L at pH of 7.0 and the biochar dose of 0.25 g at the temperature of 30°C. Figure 6(c) shows the impact of contact time on the adsorption of MO dye onto the SIPR-700. Figure 6(c) shows that, removal of MO dye was expanded with an expansion of contact time from 10 to 60 min and accomplishes the equilibrium condition at 60 min. The plausible purpose behind fast adsorption at starting stage might be because of the accessibility of increasingly number of dynamic destinations in the SIPR-700. At that point, adsorption of MO dye kept a proceeded expanding inclination, at a more slow rate pursued by a more extended balance timespan from 10 to 60 min. Further increment in contact time from 60 to 90 min, the adsorption rate turned out to be slower furthermore, no further significant adsorption has been noted.

#### Influence of biochar dosage

The impact of biochar dosage on the adsorption of MO dye was considered by varying the biochar dose from 0.05 to 0.4 g in 100 mg/L of 25 mg/L centralization of MO color arrangement at pH = 7.0 for 60 min at 30°C. Figure 6(d) shows the impact of biochar dosage on the adsorption of MO dye onto SIPR-700. It could be seen from Figure 6(d), the percentage removal of MO dye was step by step expanded with an expanding the biochar measurements—SIPR 700 from 0.05 to 0.25 g. The purpose behind this conduct is because of the accessibility of increasingly number of surface dynamic locales and bigger surface zone. Further expanding the adsorbent portion from 0.25 to 0.4 g, the percentage removal was arrived at consistent. This may be potentially because of the accomplishment of balance between the adsorbate and the adsorbent material.

#### Influence of temperature

Figure 6(e) show the percentage removal of MO dye onto SIPR-700. Adsorption study was performed by adding 0.25 g of SIPR-700 in 100 mL of 25 mg/L concentration of MO dye solution at the pH of 7.0. Figure 6(e) explored that the percentage removal of MO dye was decreased as the temperature increased from 30 to 60°C. The reason for this behavior, weak adsorptive force between the MO dye and the adsorption sites on the surface of the SIPR-700 and number of available active sites were less at higher temperature. Therefore, fewer amounts of MO dye molecules were interacting with the adsorption sites at SIPR-700. This factor could significantly affect the adsorption process by decreasing the adsorption capacity of SIPR-700. Finally, the results suggest that adsorption of MO dye on SIPR-700 was favored at lower temperature (30°C) and present adsorption process is exothermic in nature.

## Conclusion

In this investigation, the present research work concentrating on the isolation and recognizable proof of copper resistant bacteria which has potential points of interest in degrading copper present in industrial wastewater. The isolated bacteria were identified as *Pantoea dispersa* by sequencing study. The physicochemical properties of *Sphaeranthus indicus* biomass flourish with Cu(II) ions after phytoremediation and its pyrolytic subsidiaries (biochar) were explored. Plant residues acquired after phytoremediation contain a lot of Cu(II) ion. Plant residues pyrolysis at 700°C gave best conditions to the further treatment and the reutilization of plant deposits acquired after phytoremediation. This examination exhibited that pyrolysis is a successful innovation for changing over the metal(loid)-rich biomass into important items. In expansion, the pyrolysis items (SIPR-700) produced from plant residues got after phytoremediation showed incredible execution in MO dye adsorption. Consequently, pyrolysis is an attainable methodology for the transfer of plant residues got after phytoremediation also; their pyrolysis products can fill in as potential elective sorbents for the cleaning of toxic contaminants from wastewater.

## Highlights

- Rhizoremediation of Cu(II) ion contaminated soils using *Pantoea dispersa* strain with *Sphaeranthus indicus* plant has studied.
- The bioaccumulation and translocation of Cu(II) ions in *Sphaeranthus indicus* plant was estimated.
- Plant residues biochar was prepared by pyrolysis process and surface morphology of biochar was characterized.

Biosorption of methylene orange dye onto biochar has been investigated.
