# Supplementary figures and images for: Rhizoremediation of Cu(II) ions from contaminated soil using plant growth promoting bacteria: an outlook on pyrolysis conditions on plant residues for methylene orange dye biosorption
Source: Bioengineered. 2020 Feb 17;11(1):175–87. doi: 10.1080/21655979.2020.1728034 (PMC7039635; doi:10.1080/21655979.2020.1728034)

**Figure S1.** *Pantoea dispersa –* Sequence (Supplementary)

**
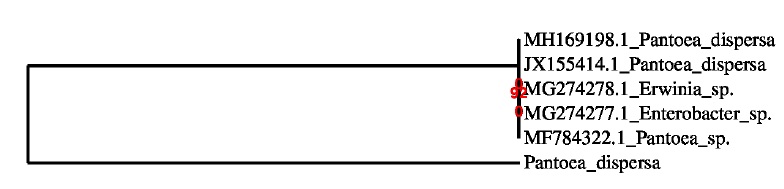
**

Supplement: Supplemental Material [file kbie-11-01-1728034-s001.docx]
